# A prediction model using 2-propanol and 2-butanone in urine distinguishes breast cancer

**DOI:** 10.1038/s41598-021-99396-5

**Published:** 2021-10-05

**Authors:** Shoko Kure, Sera Satoi, Toshihiko Kitayama, Yuta Nagase, Nobuo Nakano, Marina Yamada, Noboru Uchiyama, Satoshi Miyashita, Shinya Iida, Hiroyuki Takei, Masao Miyashita

**Affiliations:** 1 Department of Integrated Diagnostic Pathology, Nippon Medical School, 1-1-5, Sendagi, Bunkyoku, Tokyo 113-8602 Japan; 2 Department of Medicine, Mount Sinai Beth Israel, 281 First Avenue at 16th Street, New York, NY 10003 USA; 3 RIKEN KEIKI Co., Ltd., Minami-Sakaecho, Kasukabeshi, Saitama 344-0057 Japan; 4 Faculty of Medical Science, Nippon Sport Science University, 1221-1 Kamoshida-cho, Aoba-ku, Yokohama, Kanagawa 227-0033 Japan; 5 Koyukai Asakusa Clinic, 4-11-6, Asauksa, Taito-ku, Tokyo 111-0032 Japan; 6 Department of Cardiovascular Medicine, Tufts Medical Center, 800 Washington Street, Boston, MA 02111 USA; 7 Department of Breast Oncology, Nippon Medical School Chiba Hokusoh Hospital, 1715 Kamagari, Inzai, Chiba 270-1694 Japan; 8 Department of Breast Surgery and Oncology, Nippon Medical School Hospital, 1-1-5, Sendagi, Bunkyoku, Tokyo 113-8603 Japan; 9 Nippon Medical School, 1-1-5, Sendagi, Bunkyoku, Tokyo 113-8602 Japan; 10 Twin Peaks Laboratory of Medicine (TPLM), 34 Motai, Oaza, Obanazawa, Yamagata 999-4331 Japan

**Keywords:** Breast cancer, Cancer screening

## Abstract

Safe and noninvasive methods for breast cancer screening with improved accuracy are urgently needed. Volatile organic compounds (VOCs) in biological samples such as breath and blood have been investigated as noninvasive novel markers of cancer. We investigated volatile organic compounds in urine to assess their potential for the detection of breast cancer. One hundred and ten women with biopsy-proven breast cancer and 177 healthy volunteers were enrolled. The subjects were divided into two groups: a training set and an external validation set. Urine samples were collected and analyzed by gas chromatography and mass spectrometry. A predictive model was constructed by multivariate analysis, and the sensitivity and specificity of the model were confirmed using both a training set and an external set with reproducibility tests. The training set included 60 breast cancer patients (age 34–88 years, mean 60.3) and 60 healthy controls (age 34–81 years, mean 58.7). The external validation set included 50 breast cancer patients (age 35–85 years, mean 58.8) and 117 healthy controls (age 18–84 years, mean 51.2). One hundred and ninety-one compounds detected in at least 80% of the samples from the training set were used for further analysis. The predictive model that best-detected breast cancer at various clinical stages was constructed using a combination of two of the compounds, 2-propanol and 2-butanone. The sensitivity and specificity in the training set were 93.3% and 83.3%, respectively. Triplicated reproducibility tests were performed by randomly choosing ten samples from each group, and the results showed a matching rate of 100% for the breast cancer patient group and 90% for the healthy control group. Our prediction model using two VOCs is a useful complement to the current diagnostic tools. Further studies inclusive of benign tumors and non-breast malignancies are warranted.

## Introduction

Breast cancer is the most frequent cause of death in women worldwide. In 2020, over two million new cases of breast cancer were diagnosed, and 684,996 persons died from the disease^1^. The early detection of breast cancer is an important step toward achieving efficient treatment. Mammography (MG), the most commonly used screening test at present, can detect breast cancers during the asymptomatic phase and reduce mortality among women of certain ages^2–4^. Yet MG screening has several drawbacks. First, MG detects benign lesions, which can lead to unnecessary testing, treatment, and anxiety^5^. Second, MG is less sensitive in dense breast^6^. Third, MG is associated with significant pain caused by the relatively strong pressure applied to the breast. An alternative to MG that can screen for breast cancer safely, painlessly, and noninvasively is therefore urgently awaited.

Volatile organic compounds (VOCs) in biological samples such as breath and blood have been investigated in connection with cancer detection for more than two decades. The potential of VOCs as non-invasive biomarkers has been supported by reports on the capabilities of sniffer dogs and sensory devices in distinguishing between healthy controls and patients with cancers of the lung^7–10^, colon or rectum^11^, stomach^12,13^, liver^14^, head and neck^15–17^, ovaries^18^, and breast^19–26^. The combination of multiple biomarkers has strengthened the discriminatory power of this approach, raising accuracy to rates of 0.9 or higher in multiple studies.

While the previous studies have yielded promising results, critical steps still need to be taken to standardize the sample collection and storage and handling of the data, and to validate the results in independent samples. While the advantages of urine as an alternative matrix for volatile biomarkers have been outlined in lung cancer^27^, the data are scanty on cancers of other organs, including the breast. In this study we sought to identify and analyze VOCs that appear specifically in the urine of breast cancer patients. We assessed the potential of VOCs to become biomarkers of breast cancer by constructing a prediction model using a training set and an external validation set with triplicated reproducibility tests.

## Methods

### Subjects

The subjects were divided into two groups: patients with primary breast cancer and healthy volunteers (controls). The primary breast cancer patients were who were diagnosed by either fine-needle aspiration cytology or core-needle biopsy at Nippon Medical School Chiba-Hokusoh Hospital from November 2015 to October 2019 were enrolled. Clinical stages of the breast cancer patients were classified according to the Union for International Union Cancer Control (UICC) classification. The histological subtypes were based on the 15th St. Gallen International Breast Cancer Conference 2017^28^. Control subjects with no histories of previously diagnosed cancer of any type were recruited from the public in systemic cancer screenings at Nippon Medical School Chiba-Hokusoh Hospital and Koyukai Asakusa Clinic over the same period. All procedures performed in this study involving human participants were performed in accordance with the Declaration of Helsinki (as reserved in 2013). The study was approved by the ethics committees of Nippon Medical School Chiba Hokusoh Hospital (IRB#320). All subjects provided their signed informed consent before enrolment.

### Urine samples

Urine samples were collected with paper cups (Harn cup laminate A, Nissho Sangyo, Tokyo, Japan), transferred to sterile test tubes (Sterile SP tube TD4000, Eiken Chemical Co., Tokyo, Japan), sealed with caps, and stored at − 30 ℃ until analysis in 3 ml volumes. The breast cancer patients provided the samples a few days before surgery; the controls provided them during the cancer screening tests. All of the samples were transferred to the analysis institution, RIKEN KEIKI Co., Ltd., by a refrigerated courier service.

After the urine samples were thawed in a refrigerator, more than 3 mL of each sample was filtered and sterilized (Hawatch Scientific, PES Syringe Filter: Pore Size = 0.22 μm, Diameter = 25 mm, Material = PES Gama Sterile). Next, the sterilized samples were pipetted in 3 ml volumes into vials for an HS-20, and NaCl (> 99.5%, FUJIFILM Wako Pure Chemical Corporation) was added. These vials were sealed with the aluminum-cap (Silicone/PTFE, Shimazu GLC). These protocols were performed at a maximum of 3 samples at once to avoid degradation.

### Gas chromatography–mass spectrometry (GCMS)

GCMS analysis was performed with GCMS-QP2010 Ultra Gas Chromatograph Mass Spectrometer (Shimadzu Co., Kyoto, Japan) equipped with HS-20 Trap with a capillary column (Inert Cap Pure WAX, 32 m length; 0.25 mm internal diameter; 0.25 μm film thickness). A helium (99.999%) carrier gas set at a flow rate of 1.76 mL/min was used for the GCMS analysis. The GC column temperature was maintained at 30 ℃ for 5 min, raised from 30 to 250 ℃ at a rate of 10 ℃ per min, and maintained at 260 ℃ for 6 min. The mass spectrometry was performed in a scanning mode (*m*/*z* = 33.00–300.00).

### Sample and data analysis

Urine samples from breast cancer patients, healthy controls, and blank controls were analyzed in the GCMS on the same day using GCMS solution and LabSolutions Insight software Version 2.0 (Shimadzu, Co., Kyoto, Japan, https://www.shimadzu.com/an/products/liquid-chromatograph-mass-spectrometry/lc-ms-software/labsolutions-insight/index.html). The peak-data were obtained from the total ion current chromatogram (TIC) of each sample. Each set of peak-data was annotated according to the NIST/EPA/NIH Mass Spectral Library (NIST11) and WILEY REGISTRY® of Mass Spectral Data 9th Edition (Wiley9), and the area was quantitated. Air contamination and error were adjusted using a stable standard and a blank and samples. The urine of the experiment staff was used as a standard sample. Urine samples were collected from the same staff over several days and stored frozen. After a certain amount of urine has been collected, thaw the frozen urine sample in a refrigerator and mix all it to make it homogenized. After that, the sample was divided into several small-volume storage containers (Eiken Chemical FT2100 sterile screw round-bottom spits) and frozen again. These samples were used as the standard urine sample. A blank sample referred to a tube that contained room air on each day of the sample analysis. These data were further adjusted by urine creatinine levels.

The cancer urine samples were divided into two groups: a training set for building a prediction model, and an external validation set. Compounds detected in fewer than 80% of the samples from the training set were excluded from each group, and the remaining compounds were assessed by the following statistical analysis. Several of the variables were selected by stepwise analysis. Next, the compounds showing breast cancer patient (BCP) < healthy control (HC) and BCP > HC were selected. The prediction model was then constructed by discriminant analysis. The discriminant factor was analyzed by an Receiver Operating Characteristic (ROC) analysis. For reproducibility, 10 randomly chosen BCP samples and 10 randomly chosen HC samples were analyzed three times on different days. The external sample set was also tested, for validation. Discrimination analysis by multiple regression analysis was performed by Microsoft Excel (Microsoft 365). All the other statistical analyses were performed using the R statistical package (www.r-project.org).

### Ethics approval and consent to participate

This study was approved by the ethics committees of Nippon Medical School Chiba Hokusoh Hospital (IRB#320).

### Consent for publication

A signed informed consent was obtained from each participant. The informed consent is available upon request.

## Results

### Subjects enrolled in the study

Two hundred and eighty-seven subjects were enrolled in this study, including 110 BCPs and 177 HCs. The BCPs and HCs were randomly allocated to a training set and an external set using software. The training set included 60 BCPs (age 34–88 years, mean 60.3) and 60 HCs (age 34–81 years, mean 58.7). The external validation set included 50 BCPs (age 35–85 years, mean 58.8) and 117 HCs (age 18–84 years, mean 51.2) (Table 1). The clinical stages and histological subtypes are summarized in Table 1.

**Table 1:** Subjects enrolled in the study.

	Training set		External set	
	BCP	HC	BCP	HC
Number of subjects	60	60	50	117
**Age**				
Median	60 (34–88)	58.5 (34–81)	60.5 (35–85)	48 (18–84)
Mean	60.3	58.7	58.8	51.2
S.D	12.1	12.2	13.7	18.5
**Clinical stage**				
0	0	–	12	–
I	30	–	20	–
II	30	–	11	–
III	0	–	6	–
IV	0	–	1	–
**Histological subtype**				
Luminal A-like	18	–	27	–
Luminal B-like	18	–	9	–
Luminal HER2-like	8	–	4	–
Pure HER2-like	4	–	3	–
Triple-negative-like	11	–	7	–
NA	1	–	0	–

*BCP* breast cancer patient, *HC* healthy control, *NA* not applicable, *S.D.* standard deviation.

### GCMS analysis

**Figure 1:**
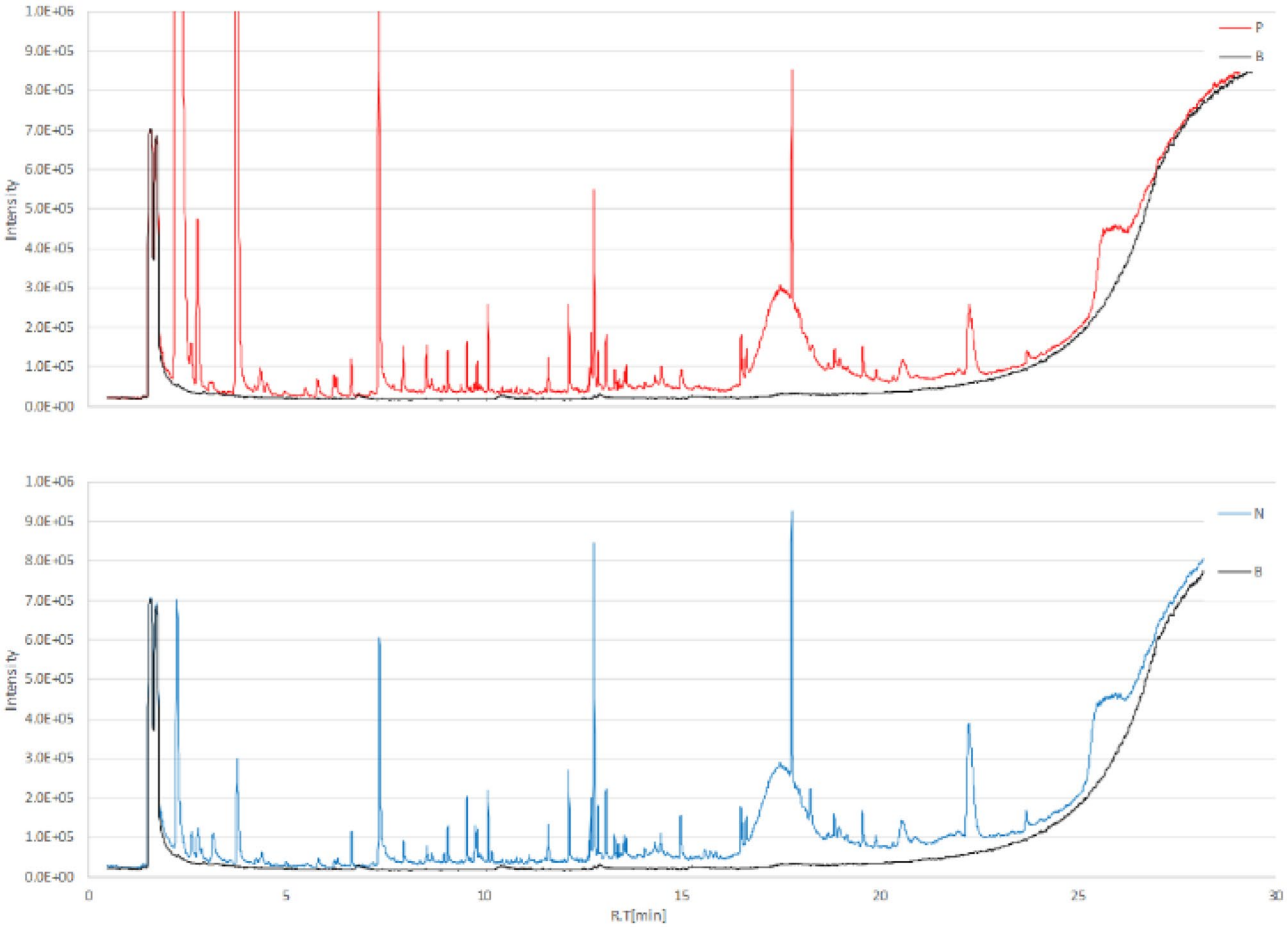
A representative GCMS total ion chromatograms (TIC) of urine volatile organic compounds. TIC of volatile organic compounds from urine samples collected from a breast cancer patient (**A**) and healthy control (**B**). Both samples showed various peaks. *TIC* total ion chromatogram.

The GCMS analysis of the urine samples showed numerous peaks. A representative total ion chromatogram (TIC) is presented in Fig. 1. Compounds detected in fewer than 80% of the subjects were excluded in each group, and 191 compounds were assessed by an ensuing statistical analysis. The compounds identified are listed in Supplemental Table S1.

### Building and validating the prediction model

**Figure 2:**
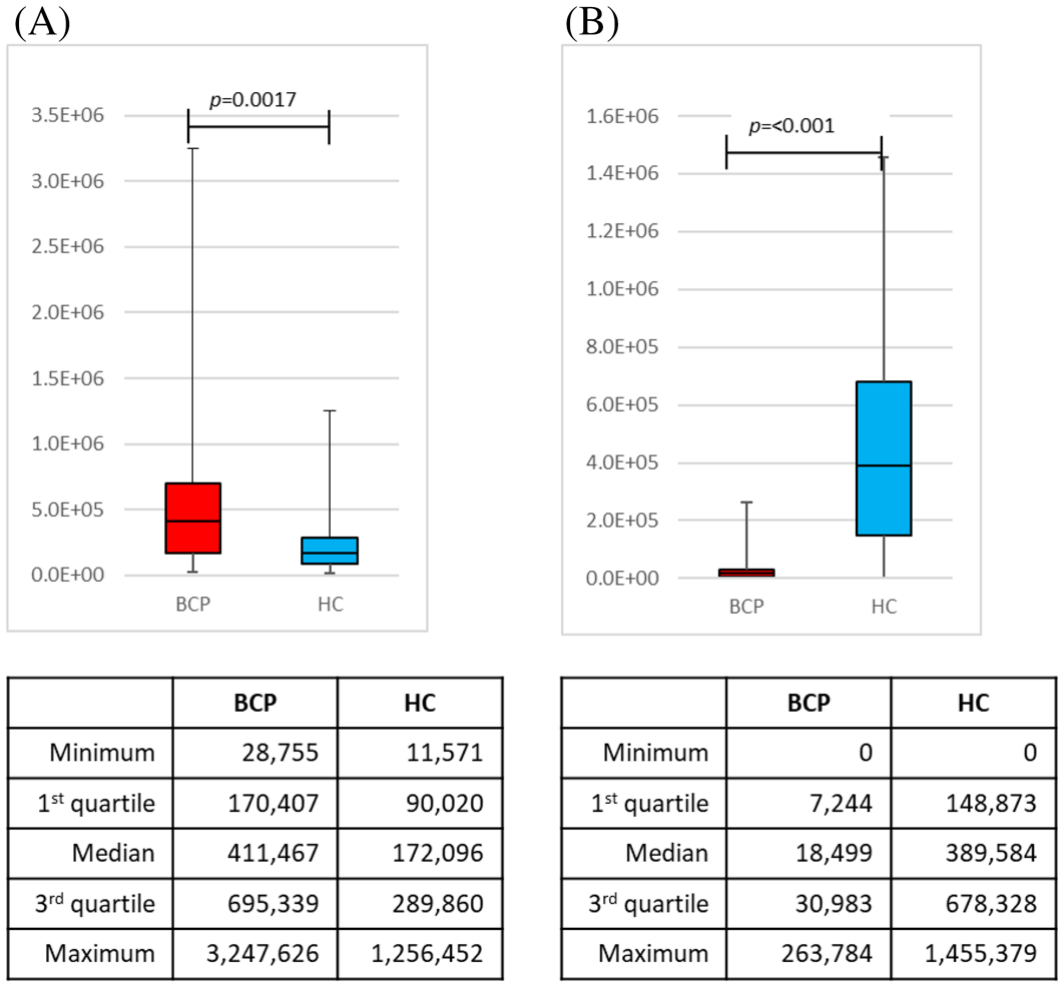
Box charts of the peak areas of the 2-butanone and 2-propanol using the model. Box plots of the peak areas of 2-propanol and 2-butanone generated by the model. The model indicated that 2-butanone was higher in breast cancer patients than in healthy controls (**A**), and that 2-propanol was higher in healthy controls than in breast cancer patients (**B**). *BCP* breast cancer patient, *HC* healthy control.

**Figure 3:**
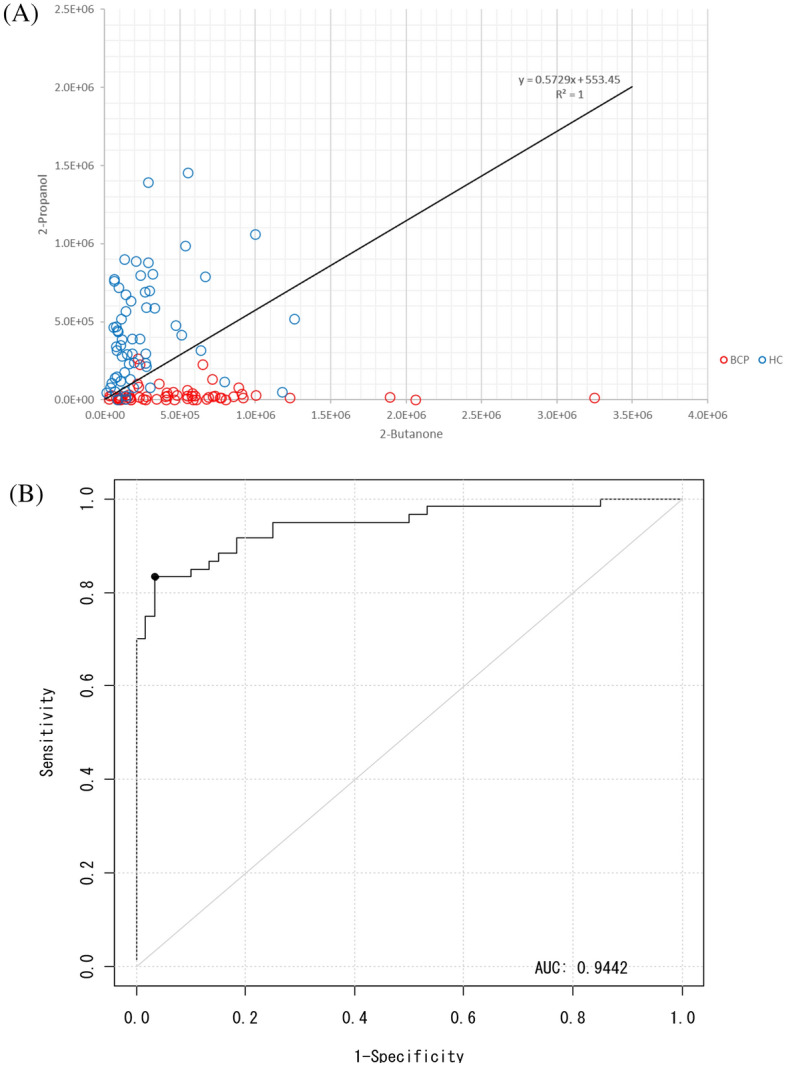
Scatter plots and the area under the curve (AUC) of the samples in the training set. (**A**) The scatter plots show the areas of 2-butanone and 2-propanol in each sample in the training set. The X-axis and Y-axis represent 2-butanone and 2-propanol, respectively. (**B**) The AUC, sensitivity, and specificity for this model were 0.9442, 93.3%, and 83.3%, respectively.

Using the detected peak data, 10 compounds were selected by a stepwise backward elimination method. Next, the prediction model was built based on the *P*-value, standardized partial regression coefficient, tolerance, and variance inflation factor (VIF). First, discriminant models were created using the area values of each compound and the area values of each compound corrected for creatinine concentration. To create the discriminant model, we used 60 samples from BCP (stage I = 30 samples, stage II = 30 samples) and 60 samples from HC. By stepwise variable selection method, the detected compounds were narrowed down to constructing the discriminant model. After narrowing down the original compound list to a few compounds, box charts of each compound were used to compare the areas between BCP and HC. The compounds which were "BCP < HC” and "BCP > HC" were selected. A linear discriminant analysis was performed using the selected compounds. The prediction model built through this procedure used two VOCs in combination, 2-propanol and 2-butanone. As the box plots generated by this model show, 2-butanone was higher in BCP than in HC, while 2-propanol was higher in HC than in BCP (Fig. 2). The obtained discriminant equation was used as the discriminant model equation. The discriminant coefficients in the discriminant model equation were obtained by ROC analysis. The scattered plot and the area under the curve (AUC) are shown in Fig. 3. Using this AUC, 0.9442, and the cutoff value were decided by Youden index. The sensitivity was 93.3%, specificity was 83.3%, positive predictive value was 84.8%, negative predictive value was 92.6%, and accuracy was 88.3% for this model (Table 2). The performance of the constructed model for the histological subtypes were also evaluated (Table 3).

**Table 2:** The performance of the constructed model with the training set.

	True condition		
	Condition positive	Condition negative	
**Inspection results**			
Inspection results positive	56	10	Positive predictive value (%)
			84.8
Inspection results negative	4	50	Negative predictive value (%)
			92.6
	Sensitivity (%)	Specificity (%)	Accuracy (%)
	93.3	83.3	88.3

**Table 3:** The results for each histological subtype when applying the model to the training set.

BCP				HC		
Subtype	True positive	False negative	Sensitivity (%)	True negative	False positive	Specificity (%)
Luminal A-like	17	1	94.4	50	10	83.3
Luminal B-like	17	1	94.4			
Luminal HER2-like	8	0	100.0			
Pure HER2-like	4	0	100.0			
Triple-negative-like	10	1	90.9			

*BCP* breast cancer patients, *HC* healthy controls.

Additional reproducibility tests were performed by randomly choosing ten models from each group in the training set. The reproducibility tests were triplicated on different days, and the results showed a matching rate of 100% for the BCP group and 90% for the HC group (Table 4).

**Table 4:** The results of the reproducibility tests.

	Name	Stage	Day 1	Day 2	Day 3	Day 1 = Day 2 = Day 3
			Result	Result	Result	Coincidence
BCP	Case 1	1	○	○	○	○
	Case 2	1	○	○	○	○
	Case 3	1	○	○	○	○
	Case 4	1	○	○	○	○
	Case 5	1	○	○	○	○
	Case 6	2	×	×	×	○
	Case 7	2	○	○	○	○
	Case 8	2	○	○	○	○
	Case 9	2	○	○	○	○
	Case 10	2	○	○	○	○
						100%
○ and × indicate true positive and false negative, respectively						
HC	Case 11	–	○	○	○	○
	Case 12	–	×	×	×	○
	Case 13	–	○	○	○	○
	Case 14	–	○	○	○	○
	Case 15	–	○	○	×	×
	Case 16	–	○	○	○	○
	Case 17	–	○	○	○	○
	Case 18	–	○	○	○	○
	Case 19	–	×	×	×	○
	Case 20	–	○	○	○	○
						90%
○ and × indicate true negative and false positive, respectively						

*BCP* breast cancer patients, *F* female HC, healthy controls.

### Validation test using an external validation set

**Figure 4:**
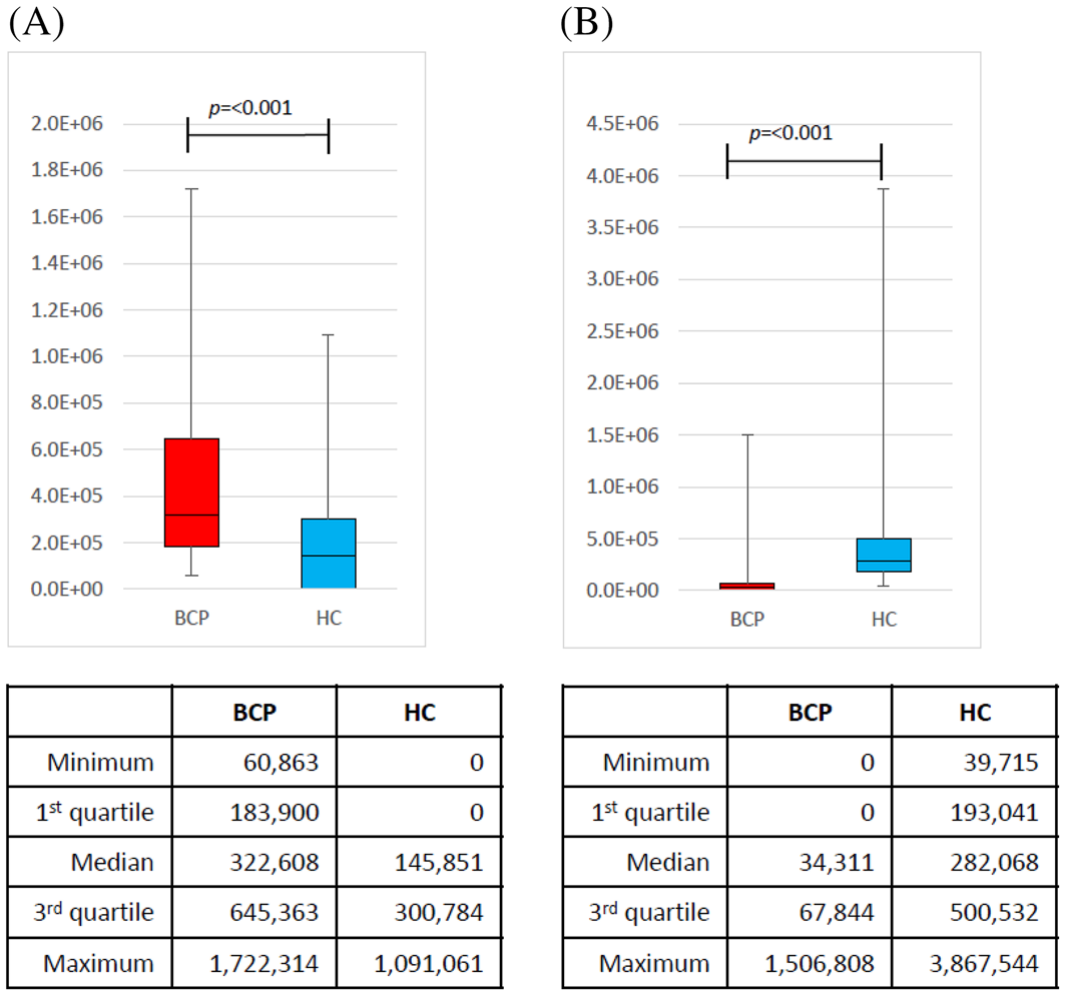
Box charts of the peak areas of the 2-butanone and 2-propanol of the external validation set using the model. Box plots of the peak areas of 2-propanol and 2-butanone in the external validation set using the model. The peak areas in the external validation set were similar to those in the training set. (**A**) Peak areas of 2-butanone and (**B**) 2-propanol. *BCP* breast cancer patient, *HC* healthy control.

**Figure 5:**
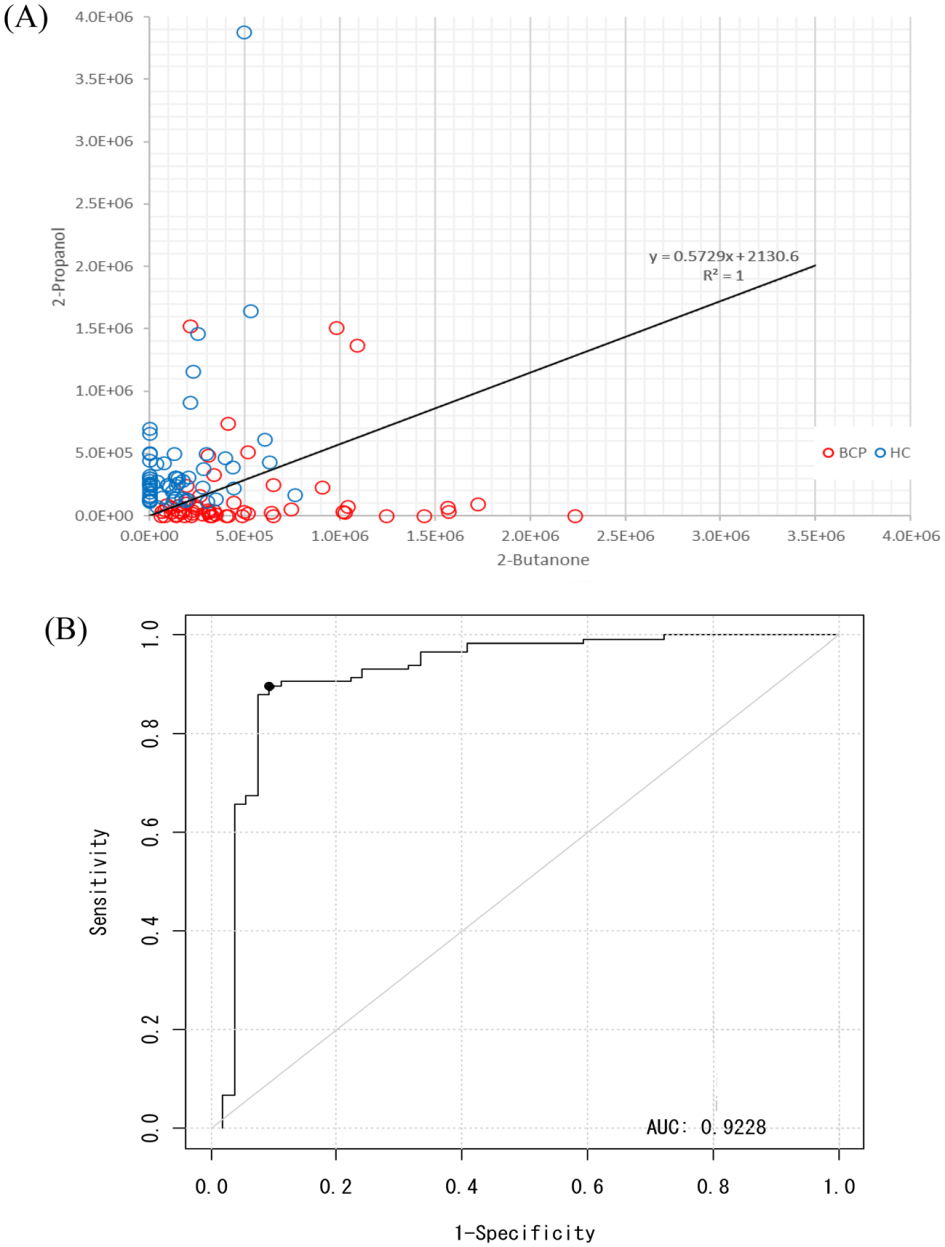
Scatter plots of the samples and the area under the curve (AUC) in the external validation sets. (**A**) The external validation set was used to confirm the validity of the training models. (**B**) The area under the curve using the external validation set. The AUC, sensitivity, and specificity for this model were 0.9228, 84.0%, and 90.6%, respectively.

Next, an external validation set was used to confirm the validity of the training models. The box plots of the peak areas of 2-butanone and 2-propanol generated by the constructed model are shown in Fig. 4. The AUC was 0.9228, sensitivity was 84%, specificity was 90.5%, positive predictive value was 79.2%, negative predictive value was 92.9%, and accuracy was 88.6% (Fig. 5 and Tables 5, 6).

**Table 5:** The performance of the constructed model applying to the external validation set.

	True condition		
	Condition positive	Condition negative	
**Inspection results**			
Inspection results positive	42	11	Positive predictive value (%)
			79.2
Inspection results negative	8	105	Negative predictive value (%)
			92.9
	Sensitivity (%)	Specificity (%)	Accuracy (%)
	84.0	90.5	88.6

*BCP* breast cancer patient, *HC* healthy control.

**Table 6:** The results for each histological subtype when applying the model to the external validation set.

BCP				HC		
Subtype	True Positive	False Negative	Sensitivity (%)	True Negative	False Positive	Specificity (%)
Luminal A-like	22	5	81.5	105	11	90.5
Luminal B-like	8	1	88.9			
Luminal HER2-like	3	1	75.0			
Pure HER2-like	2	1	66.7			
Triple-negative-like	7	0	100.0			

*BCP* breast cancer patient, *HC* healthy control.

## Discussion

To the best of our knowledge, our study is the first to build a prediction model to detect breast cancer using a combination of only two VOCs, with the results validated by triplicated reproducibility tests and a validation set analyzed by GCMS. Among the different types of volatile compounds analyzed, we demonstrated that a combination of 2-butanone and 2-propanol was highly effective in detecting breast cancer at various clinical stages, achieving a sensitivity of 93.3% and a specificity of 83.3%.

While cancer screening biomarkers using biological samples have been extensively for the past decade, including several with blood and urine, a model to detect specific VOCs as cancer biomarkers holds the potential for application as a rapid, noninvasive, and inexpensive cancer screening technique that reduces the burdens on the individuals screened. The science of detecting cancers through body fluids was pioneered by Linus Pauling in 1971^29^. More recently, the development of sensor techniques and devices has led to an exponential increase in studies to detect cancer from samples of exhaled breath, blood, urine, and cell-cultured mediums of cancers of the the lung^7–10^, colon or rectum^11^, stomach^12,13^, liver^14^, head and neck^15–17^, prostate^30^, kidney^31^, ovaries^18^, and breast^19–26^. Most of the previous studies on VOCs in breast cancer patients have examined breath samples. A series of studies by Phillips et al.^25,32^ demonstrated that breath oxidative stress markers can distinguish between women with breast cancer and healthy controls. Later, experiments by the same group found that breath samples show good potential as a biomarker for breast cancer^21^. Compared with earlier studies that included both early and advanced stages of breast cancer, our study is unique in enrolling a large population mainly consisting of patients with early-stage breast cancer, a disease difficult to diagnose with the current screening methods. Our study design supports the utility of this screening for early-stage breast cancer.

While breath sampling is easy and non-invasive, several obstacles impede further research on the development of the technique for cancer screening^33^. The procedure is impracticable for routine application, the samples are unstable, and noise from background concentrations can interfere with the very small concentrations of volatiles involved. Urine samples are thus expected to offer an alternative matrix for detecting VOC biomarkers. Adapting the headspace gas of urine samples to GCMS analysis has been well established using classical and basic water analysis techniques. Further, the urine can be partitioned, dispensed, mixed, spikes, stored, and dispatched. The only previous studies to investigate VOCs in urine samples of breast cancer patients were based on smaller populations and employed neither reproducibility tests nor external validation sets^34–36^. Our study sought to build a VOC-screening model with urine samples using a training set, an external set, and triplicated reproducublity tests.

The previous studies on the breath samples of breast cancer patients investigated analyses with cross-validation tests^20,21,25^ and/or external validation sets^21^. We designed and performed triplicated reproducibility tests by randomly choosing ten samples from each group as a training set and then confirmed the results with an external validation set. The results showed a concordance of 100% for the BCP group and 90% for the HC group, and the constructed model was confirmed by the external validation set. This is the first study to perform triplicated reproducibility tests and validation tests with external sets.

No studies to date have a identified a single VOC that can determine the presence of cancer with high reliability, and no consensus has been reached on a causative connection between specific VOCs or any type of cancer. A number candidate VOCs have the potential to become common biomarkers among cancers in general, but none are specific to any one type of cancer. A combination of two different VOCs in breath might serve as a marker of disease when one is high and the other is low^22^. We therefore postulated that combinations of several VOCs may be able to increase the overall sensitivity and specificity.

Several previous studies on VOCs in breast cancer patients are listed in Table 7. Previous analyses of the urine samples of breast cancer patients^34–36^ have identified a significant decrease in dimethyl disulfide, and increases in 4-carene, 3-heptanone, phenol, 1,2,4-trimethylbenzene, and 2-methoxythiophene. In our samples, the combination of 2-butanone and 2-propanol was the best detector of breast cancer of various clinical stages, achieving a sensitivity and specificity of 93.3% and 83.3%, respectively. The VOC 2-propanol, also known as isopropyl alcohol, is a colorless liquid used in making cosmetics, perfumes, skin and hair products, and other chemicals. Elevated levels of 2-propanol were identified in an earlier breath analysis of breast cancer patients^22^ and a study of lung cancer cells in vivo^37^. On the other hand, 2-propanol was significantly decreased VOCs in urine samples of cholangiocarcinoma or pancreatic cancer^38^. The level of 2-propanol may change in association with the altered activity of cytochrome P450 in breast cancer^26^, and the discrepancy of the result may attribute to the different sample types (i.e. breath or urine) used in each study. The detailed mechanism needs to be clarified. The VOC 2-butanone, more widely known as methyl ethyl ketone, is a widely used solvent. It can be obtained by the dehydration of 2,3-butanediol, a natural metabolite produced from glucose by several microorganisms such as *Escherichia coli* and *Klebsiella pneumoniae*^39–41^. Our preliminary test confirmed that 2-butanone was not eluted from blank tubes (data not shown). Several ketones, including 2-butanone, are elevated in the breath samples of lung cancer patients^42^, urine samples of prostate cancer patients^43^, and one study identified higher levels of 2-butanone in cultured lung cell lines than in normal cell lines in vitro^37^. The possible origins are endogenous production, microbiota, or environmental exposure. Fatty acid oxidation, the mechanism found to cause 2-butanone production in cancer progression, may result in elevated levels of ketones^44,45^. Butanoate metabolism is also reported to be highly activated in breast cancer and colon cancer patients^34^. Though 2-butanone has not been identified as a specific marker of breast cancer, the combination of 2-propanol and 2-butanone, applied with our prediction model constructed by multivariate analysis, proved to be extremely sensitive and specific in distinguishing breast cancer of all histological subtypes from healthy controls. Our study is the first to build a prediction model based on the *P*-value, standardized partial regression coefficient, and VIF. Further studies to identify the underlying biological mechanism of this combination of VOCs, and its clinical significance for daily practice, are merited.

**Table 7:** Published studies on VOCs on breast cancer.

Authors	Sample	Methods, results
Phillips et al. (2003)^25^	Breath	GCMS, methylated alkane contour
	BC (51) vs abnormal MG (50)	Sensitivity 62.7%(32/51), specificity 84.0% (42/50)
	BC (51) vs healthy (42)	Sensitivity 94.1% (48/51), specificity 73.8% (31/42)
Phillips et al. (2006)^22^	Breath (re-analysis of ref.#24)	GCMS
	BC (51) vs abnormal MG (50)	2-propanol, 2,3-dihydro-1-phenyl-4(1H)-quinazolinone,
	BC (51) vs healthy (42)	1-phenyl-ethanone, heptanal, and isopropyl myristate
		Sensitivity 93.8%, specificity 84.6%
Phillips et al. (2010)^24^	Breath	GCMS
	BC (54) vs healthy (204)	Training set: Sensitivity 78.5%, specificity 88.3%
		Test set: sensitivity 75.3%, specificity 84.8%
Patterson et al. (2011)^48^	Breath	GCMS
	BC (20) vs healthy (20)	Sensitivity 72%, specificity 64%
Silva et al. (2012)^36^	Urine	GCMS
	BC (26) vs healthy (21)	↓dimethyl disulfide
		↑4-carene, 3-heptanone, phenol,
		1,2,4-trimethylbenzene, 2-methoxythiophene,
		Sensitivity/Specificity NA
Mangler et al. (2012)^26^	Breath	GCMS
	BC (10) vs healthy (10)	↓3-methylhexane, decene, caryophyllene, naphthalene
		↑trichlorethylene
		Sensitivity/Specificity NA
Li et al. (2014)^46^	Breath	GCMS
	BC (22) vs healthy (24)	Hexanal, heptanal, octanal,
	vs Breast benign tumor (17)	and nonanal,
		Sensitivity 72.7%, specificity 91.7%
Wang et al. (2014)^47^	Breath	GCMS
	BC (39) vs healthy (45)	2,5,6-trimethyloctane,
	vs cyclomastopathy (25)	1,4-dimethoxy-2,3-butanediol, cyclohexanone
	vs mammary gland fibroma (21)	Sensitivity/specificity NA
Barash et al. (2015)^20^	Breath	GCMS
	BC (90) vs benign (13) vs healthy (23)	23 compounds
		Sensitivities 81–88%, specificities 76–96%
Silva et al. (2017)^49^	BC cell lines	GCMS
		2-Pentanone, 2-heptanone, 3-methyl-3-buten-1-ol,
		ethyl acetate,
		ethyl propanoate and 2-methyl butanoate
		Sensitivity/Specificity NA
Phillips et al. (2017)^19^	Breath	GCMS
	BC (54) vs healthy (214)	21 compounds,
		Training set: AUC = 0.79,
		Test set: AUC = 0.77
Cavaco (2018)^50^	Saliva	GCMS
	BC (66) vs healthy (40)	3-methyl-pentanoic acid, 4-methyl-pentanoic acid,
		phenol, acetic acid, propanic acid, 1,2-decanediol
		Sensitivity/specificity NA
Porto-Figueira et al. (2018)^34^	Urine	Needle Trap Microextraction (NTME)/GCMS
	BC vs healthy	2-bromophenol, octanoic acid, phenol,
		Sensitivity/specificity NA
Phillips et al. (2018)^21^	Breath	-GCMS: test accuracy = 90%
	BC (54) vs healthy (124)	-GC-surface acoustic wave detection (GCSAW): test accuracy = 86%
Silva (2019)^35^	Urine	GCMS
	BC (31) vs healthy (40)	10 compounds (sulfur compounds, terpenoids and
		carbonyl compounds),
		Sensitivity/Specificity NA, AUC = 0.842
de Leon-Martinez et al. (2020)^51^	Breath	“Electrical nose”, Compounds NA,
	BC (262) vs healthy (181)	Sensitivity 100%, specificity 100%
Zhang et al. (2020)^52^	Breath	GCMS, combination of (*S*)‐1,2‐propanediol,
	BC (78) vs healthy (71)	cyclopentanone, ethylenecarbonate, 3‐methoxy‐1,2
	vs gastric cancer (54)	propanediol, 3‐methylpyridine, phenol,
		and tetramethylsilane
		Sensitivity 93.36%, specificity 71.6%

*BC* breast cancer, *GCMS* gas chromatography–mass spectrometry, *NA* not applicable, *AUC* area under the curve.

Our study has some limitations. First, the results were derived from an analysis of a fairly non-diverse population, and thus may not extend to a broader population. Second, the control samples were only collected from healthy individuals, and the VOCs examined were not confirmed to be breast-cancer-specific as biomarkers. Previous analyses of VOCs in breath samples^20,46,47^ have shown that, among the VOCs that were significantly increased in breast cancer patients versus healthy controls and benign breast tumors, only one compound was significantly altered in the breast cancer patients versus the benign tumors. While the two markers in the current study are useful in complementing the current diagnostic tools, further studies with larger populations inclusive of benign tumors and non-breast malignancies are warranted. Furthermore, since breast cancer is a heterogeneous disease, analysis including the molecular subtypes, which is an independent classification from the histological subtypes, is desirable. However, the molecular subtypes were not available for the current study. To further substantiate our results, molecular subtypes by gene expression analysis are needed.

## Conclusion

Our prediction model using the combination of the VOCs 2-propanol and 2-butanone usefully complements the current diagnostic tools for early-stage breast cancer. Further studies inclusive of benign tumors and non-breast malignancies are warranted.

## Supplementary Information

Supplementary Table S1.

## Data Availability

The datasets used and/or analyzed during the current study are available from the corresponding author, for reasonable uses, upon request.

## Abbreviations

AUC: Area under the curve
BCP: Breast cancer patient
GCMS: Gas chromatography–mass spectrometry
HC: Healthy control
MG: Mammography
NA: Not applicable
ND: Not determined
ROC: Receiver operating characteristic
S.D.: Standard deviation
TIC: Total ion current chromatogram
VIF: Variance inflation factor
VOC: Volatile organic compound
