# Supplementary material for: A prediction model using 2-propanol and 2-butanone in urine distinguishes breast cancer
Source: Sci Rep. 2021 Oct 5;11:19801. doi: 10.1038/s41598-021-99396-5 (PMC8492640; doi:10.1038/s41598-021-99396-5)
Supplement: Supplementary file 1 — Supplementary Table S1. [file 41598_2021_99396_MOESM1_ESM.docx]

Supplement Table S1. List of the identified VOCs (detection rate>80%).

| No. | Name | R.T. |
| --- | --- | --- |
| 1 | Propane, 1-chloro- | 2.047 |
| 2 | Oxirane, 2,2-dimethyl- | 2.060 |
| 3 | Propanal | 2.170 |
| 4 | 2-Propanone | 2.260 |
| 5 | 2 methyl tetrahydrofuran 3 one | 2.603 |
| 6 | Methacrolein | 2.623 |
| 7 | Furan, 2,5-dihydro | 2.630 |
| 8 | 2-Butenal | 2.643 |
| 9 | Ethyl Acetate | 2.703 |
| 10 | 2-Butanone | 2.790 |
| 11 | Cyclotrisiloxane, hexamethyl- | 2.853 |
| 12 | Butanal, 2-methyl- | 2.923 |
| 13 | Butanal, 3-methyl- | 2.970 |
| 14 | 2-Butanone, 3-methyl- | 3.083 |
| 15 | 2-Propanol | 3.153 |
| 16 | 2,4-Hexadiyne | 3.173 |
| 17 | Pentane, 1-chloro- | 3.290 |
| 18 | Trisiloxane, octamethyl- | 3.433 |
| 19 | Furan, 2,5-dimethyl- | 3.443 |
| 20 | Oxirane, 2-methyl-2-propyl- | 3.577 |
| 21 | 2,4-Dimethylfuran | 3.623 |
| 22 | 2-Pentanone | 3.777 |
| 23 | Propanamide, N-(aminocarbonyl)- | 3.830 |
| 24 | Heptane, 2,2,4,6,6-pentamethyl- | 4.267 |
| 25 | 2-Pentanone, 4-methyl- | 4.387 |
| 26 | 2-Pentanone, 3-methyl- | 4.530 |
| 27 | Formic acid, butyl ester | 4.747 |
| 28 | Benzene, methyl- | 5.000 |
| 29 | Pentane, 3,3-diethyl- | 5.363 |
| 30 | 3-Hexanone | 5.510 |
| 31 | 2,3,5-Trimethylfuran | 5.693 |
| 32 | Disulfide, dimethyl | 5.827 |
| 33 | 3-Hexanone, 4-methyl- | 6.153 |
| 34 | Acetic acid, hexyl ester | 6.227 |
| 35 | 2-Hexanone | 6.237 |
| 36 | Cyclobutanol, 1-butyl- | 6.273 |
| 37 | Hexanal | 6.347 |
| 38 | 4-Methyl-4-pentene-2-one (or 3-hexene-2,5-diol) | 6.397 |
| 39 | Cyclotetrasiloxane, octamethyl- | 6.667 |
| 40 | Butanal, 2-ethyl- | 6.870 |
| 41 | 2-Pentanol, 2-methyl- | 7.050 |
| 42 | 1-Pentanol, 3-methyl- | 7.143 |
| 43 | pentadecane | 7.143 |
| 44 | Cis-methyl propenyl ketone | 7.170 |
| 45 | Ethylbenzene | 7.200 |
| 46 | 2-Propanol, 1-methoxy- | 7.283 |
| 47 | 4-Heptanone | 7.370 |
| 48 | p-Xylene | 7.493 |
| 49 | 2-Hexanone, 5-methyl- | 7.727 |
| 50 | Propanoic acid, butyl ester | 7.857 |

| 51 | Propanoic acid, 2-hydroxy-2-methyl-, ethyl ester | 7.917 |
| --- | --- | --- |
| 52 | 3-Heptanone | 7.977 |
| 53 | Propanoic acid, 2,2-dimethyl-, pentyl ester | 8.150 |
| 54 | l-Phellandrene | 8.220 |
| 55 | cyclopentanone | 8.263 |
| 56 | Benzene, 1,2-dimethyl- | 8.390 |
| 57 | .alpha.-Terpinene | 8.520 |
| 58 | 2-Propenoic acid, butyl ester | 8.530 |
| 59 | 2-Heptanone | 8.570 |
| 60 | Heptanal | 8.633 |
| 61 | Hexanal, 2-ethyl- | 8.700 |
| 62 | Tetrasiloxane, decamethyl- | 8.797 |
| 63 | (+)-3-Methylcyclopentanone | 8.877 |
| 64 | Benzene, chloro- | 8.943 |
| 65 | Oxirane, [(1-methylethoxy)methyl]- | 8.980 |
| 66 | Eucalyptol (1,8-cineole) | 9.003 |
| 67 | trans-Sabinene hydrate | 9.027 |
| 68 | 2-Heptanone, 4-methyl- | 9.100 |
| 69 | (+-)-frontalin | 9.163 |
| 70 | 2(3H)-Furanone, 4,5-dihydro-4-(2,3-dimethyl-2-buten-4-yl)- | 9.177 |
| 71 | Benzene, (1-methylethyl)- | 9.233 |
| 72 | Disulfide, methyl propyl | 9.393 |
| 73 | butanoic acid, butyl ester | 9.437 |
| 74 | Diisodecyl ether | 9.540 |
| 75 | Dodecane | 9.557 |
| 76 | Silane, (4-methoxyphenyl)trimethyl- | 9.580 |
| 77 | 2-Heptanone, 6-methyl- | 9.673 |
| 78 | Hexane, 2,3,4-trimethyl- | 9.773 |
| 79 | Dodecane, 1-chloro- | 9.780 |
| 80 | .gamma.-Terpinene | 9.780 |
| 81 | Styrene | 9.793 |
| 82 | Hexane, 2,3,4-trimethyl- | 9.797 |
| 83 | Carbonic acid, allyl hexyl ester | 9.800 |
| 84 | 2-Heptanone, 4,6-dimethyl- | 9.867 |
| 85 | 4,8-Dimethyl-nona-3,8-dien-2-one | 9.907 |
| 86 | Benzene, 1-ethyl-2-methyl- | 9.910 |
| 87 | Decane, 5,6-dimethyl- | 9.920 |
| 88 | unidentified C3-benzene | 9.937 |
| 89 | 4-Oxononanal | 9.970 |
| 90 | Cyclopentasiloxane, decamethyl- | 10.117 |
| 91 | o-Cymene | 10.167 |
| 92 | Cyclohexanone | 10.213 |
| 93 | Benzene, 1,2,3-trimethyl- | 10.233 |
| 94 | 2,4-Pentanedione, 3-methyl- | 10.387 |
| 95 | Octane, 2,7-dimethyl- | 10.460 |
| 96 | vinyl-2,6,8-trimethyl-4-nonyl ether | 10.463 |
| 97 | 3-Hexanol, 2,3-dimethyl- | 10.473 |
| 98 | 4-Heptanol, 4-methyl- | 10.503 |
| 99 | Nonadecane | 10.597 |
| 100 | 3-Octanone | 10.613 |
| 101 | 3-Heptanol, 3-methyl- | 10.703 |
| 102 | 2-Butanethiol, 2,3-dimethyl- | 10.783 |
| 103 | 2-Nonanone | 10.830 |
| 104 | 2-(Trimethylacetyl)thiophene | 10.837 |
| 105 | 4-Heptanol, 2,4-dimethyl- | 10.943 |
| 106 | 3-Ethylcyclopentanone | 10.967 |
| 107 | Methylthio-2-propanone | 11.040 |
| 108 | Benzene, 1,4-diethyl- | 11.070 |
| 109 | Butanoic acid, 2-hydroxy-, methyl ester | 11.090 |
| 110 | Benzene, 1-propenyl- | 11.117 |
| 111 | 3-Octanone, 2-methyl- | 11.193 |
| 112 | 2-Octanol, 2-methyl- | 11.237 |
| 113 | 6-Methyl-5-hepten-2-one | 11.283 |
| 114 | 6-Tetradecanone | 11.433 |
| 115 | 5-Ethyl-2-heptanone | 11.453 |
| 116 | 2-Pentanone, 4-hydroxy-4-methyl- | 11.507 |
| 117 | Allyl Isothiocyanate | 11.560 |
| 118 | 1,3-Pentadiene, 2,4-di-t-butyl- | 11.570 |
| 119 | Isobutyl-2-heptenone | 11.580 |
| 120 | 1H-Indene, 2,3-dihydro- | 11.583 |
| 121 | Formic acid, 2-ethylhexyl ester | 11.633 |
| 122 | Dimethyl trisulfide | 11.717 |
| 123 | 2,6-Dimethyloctan-2-ol | 11.753 |
| 124 | 4-Octanol, 4-methyl- | 11.927 |
| 125 | Citronellol epoxide (R or S) | 12.040 |
| 126 | 2-Isopropyl-5-methyl-1-heptanol | 12.043 |
| 127 | 3-Propylglutaric acid | 12.050 |
| 128 | 3-Heptanone, 4-methyl- | 12.060 |
| 129 | Acetic acid, 2-ethylhexyl ester | 12.157 |
| 130 | Benzene, 1-pentenyl- | 12.357 |
| 131 | 2-Pentadecanone | 12.380 |
| 132 | 3-Buten-2-one, 4-(2-hydroxy-2,6,6-trimethylcyclohexyl)- | 12.493 |
| 133 | 3-Heptyne-2,6-dione, 5-methyl-5-(1-methylethyl)- | 12.657 |
| 134 | 1,2-Bis(.gamma.-trimethylsilypropoxy)ethane | 12.717 |
| 135 | Acetic acid | 12.760 |
| 136 | Benzene, 1,3-bis(1,1-dimethylethyl)- | 12.793 |
| 137 | 4-hydroxy-1,8-cineole | 12.833 |
| 138 | 6-Dodecanone | 12.903 |
| 139 | 2-Ethyl-1-hexyl propionate | 13.087 |
| 140 | Sulfurous acid, nonyl pentyl ester | 13.303 |
| 141 | 3-Pentanone, 2,2,4,4-tetramethyl- | 13.343 |
| 142 | Dodecan - one | 13.410 |
| 143 | 2-Undecanone | 13.467 |
| 144 | 2-Ethylhexyl acrylate | 13.543 |
| 145 | 1H-Pyrrole | 13.613 |
| 146 | Camphor | 13.667 |
| 147 | Benzaldehyde | 13.697 |
| 148 | 3-Octanol, 3-ethyl- | 13.887 |
| 149 | 2-Pentadecanone, 6,10,14-trimethyl- | 14.070 |
| 150 | n-Butyric acid 2-ethylhexyl ester | 14.117 |
| 151 | Epoxy-linalooloxide | 14.193 |
| 152 | Cyclopentylacetone | 14.213 |
| 153 | 3-tert-Butyl-2-pyrazolin-5-one | 14.323 |
| 154 | Propanoic acid, 2-methyl- | 14.357 |
| 155 | 3-tert-Butyl-2-pyrazolin-5-one | 14.480 |
| 156 | Cyclohexanol, 3-(acetyloxymethyl)-2,2,4-trimethyl- | 14.657 |
| 157 | 3-Cyclohexen-1-ol, 4-methyl-1-(1-methylethyl)- | 14.900 |
| 158 | 3,4-Octanedione | 14.980 |
| 159 | trans-5-Isopropyl-6,7-epoxy-8-hydroxy-8-methylnonan-2-one | 15.127 |
| 160 | 2,4-Pentanedione | 15.243 |
| 161 | Benzene, (1,1-dimethylpropyl)- | 15.423 |
| 162 | 5,7-Dioxooctanoic acid | 15.620 |
| 163 | 2,4,7,9-Tetramethyl-5-decyn-4,7-diol | 15.753 |
| 164 | Tetradecane, 5-methyl- | 15.877 |
| 165 | Butane, 2-iodo-3-methyl- | 16.007 |
| 166 | Azulene | 16.320 |
| 167 | 1H-Indene, octahydro-2,2,4,4,7,7-hexamethyl-, trans- | 16.537 |
| 168 | Benzene, 1,3-bis(1,1-dimethylethyl)-5-methyl- | 16.583 |
| 169 | 3-tert-Butyl-2-pyrazolin-5-one | 16.643 |
| 170 | 2-Propyl-1-pentanol | 17.060 |
| 171 | Benzenamine, 2,6-dimethyl- | 17.560 |
| 172 | 2,4,6-Triethyl-5-propylcyclohex-2-en-1-one | 17.790 |
| 173 | Methane, sulfonylbis- | 17.927 |
| 174 | 2-Pentanone, 4-methyl-4-phenyl- | 18.127 |
| 175 | Benzyl alcohol, .alpha.-isobutyl-2,4,5-trimethyl- | 18.127 |
| 176 | p-n-Butylacetophenone | 18.193 |
| 177 | Pentan-1,3-dioldiisobutyrate,2,2,4-trimethyl - | 18.260 |
| 178 | Hexanoic acid, 2-ethyl- | 18.717 |
| 179 | 8,8,9-Trimethyl-deca-3,5-diene-2,7-dione | 18.870 |
| 180 | Heptanoic acid, anhydride | 18.930 |
| 181 | 3-Octen-2-one, 4-methoxy- | 18.973 |
| 182 | Silane, trimethylphenoxy- | 19.057 |
| 183 | Heptanoic acid, anhydride | 19.080 |
| 184 | Phenol | 19.190 |
| 185 | (+)-Longicamphenylone | 19.567 |
| 186 | 5-acetyl-2-dihydro-6-methyl-2-oxo-4-phenyl-3-pyridinecarbonitrile | 19.953 |
| 187 | p-Cresol | 20.000 |
| 188 | Cyclohexanol, 2-(1,1-dimethylethyl)- | 20.387 |
| 189 | Phenol, 4-(1,1-dimethylethyl)- | 21.990 |
| 190 | Phenol, 2,4-bis(1,1-dimethylethyl)- | 22.263 |
| 191 | 3,5-di-tert-Butyl-4-hydroxybenzaldehyde | 23.713 |

R.T., retention time.
